# Autophagic and Proteasomal Mediated Removal of Mutant Androgen Receptor in Muscle Models of Spinal and Bulbar Muscular Atrophy

**DOI:** 10.3389/fendo.2019.00569

**Published:** 2019-08-20

**Authors:** Maria Elena Cicardi, Riccardo Cristofani, Valeria Crippa, Veronica Ferrari, Barbara Tedesco, Elena Casarotto, Marta Chierichetti, Mariarita Galbiati, Margherita Piccolella, Elio Messi, Serena Carra, Maria Pennuto, Paola Rusmini, Angelo Poletti

**Affiliations:** ^1^Dipartimento di Scienze Farmacologiche e Biomolecolari (DiSFeB), Dipartimento di Eccellenza 2018-2022, Centro di Eccellenza sulle Malattie Neurodegenerative, Università degli Studi di Milano, Milan, Italy; ^2^Dipartimento di Scienze Biomediche, Metaboliche e Neuroscienze, Centro Interdipartimentale di Neuroscienze e Neurotecnologie (CfNN), Università degli Studi di Modena e Reggio Emilia, Modena, Italy; ^3^Department of Neurosciences, Neuromuscular Center, University of Padova, Padova, Italy; ^4^Department of Biomedical Sciences, University of Padova, Padova, Italy; ^5^Dulbecco Telethon Institute, Centre for Integrative Biology (CIBIO), University of Trento, Povo, Italy; ^6^Centro InterUniversitario sulle Malattie Neurodegenerative, Università degli Studi di Firenze, Milan, Italy

**Keywords:** autophagy, chaperones, protein aggregation, androgen receptor, motoneuron disease

## Abstract

Spinal and bulbar muscular atrophy (SBMA) is an X-linked motoneuron disease (MND) caused by a mutant androgen receptor (AR) containing an elongated polyglutamine (polyQ) tract. ARpolyQ toxicity is triggered by androgenic AR ligands, which induce aberrant conformations (misfolding) of the ARpolyQ protein that aggregates. Misfolded proteins perturb the protein quality control (PQC) system leading to cell dysfunction and death. Spinal cord motoneurons, dorsal root ganglia neurons and skeletal muscle cells are affected by ARpolyQ toxicity. Here, we found that, in stabilized skeletal myoblasts (s-myoblasts), ARpolyQ formed testosterone-inducible aggregates resistant to NP-40 solubilization; these aggregates did not affect s-myoblasts survival or viability. Both wild type AR and ARpolyQ were processed via proteasome, but ARpolyQ triggered (and it was also cleared via) autophagy. ARpolyQ reduced two pro-autophagic proteins expression (BAG3 and VCP), leading to decreased autophagic response in ARpolyQ s-myoblasts. Overexpression of two components of the chaperone assisted selective autophagy (CASA) complex (BAG3 and HSPB8), enhanced ARpolyQ clearance, while the treatment with the mTOR independent autophagy activator trehalose induced complete ARpolyQ degradation. Thus, trehalose has beneficial effects in SBMA skeletal muscle models even when autophagy is impaired, possibly by stimulating CASA to assist the removal of ARpolyQ misfolded species/aggregates.

## Introduction

Spinal and bulbar muscular atrophy (SBMA) is an inherited X-linked motoneuron disease (MND) linked to a CAG triplet repeat expansion present in the exon 1 of the gene coding for the androgen receptor (AR) (1). Because of that, the AR protein carries an elongated polyglutamine (polyQ) tract in its N-terminus. In normal individuals, the polyQ tract is comprised between 9 and 37 Qs with an average value of 22, but SBMA patients have a polyQ tract longer than 38 Qs (ARpolyQ) with a maximum of 68 Qs observed so far in some patients affected by a pathology characterized by an unusual early onset (2, 3). The physiological role of the polyQ tract is still largely debated, but the region could act as a transcriptional regulatory domain (4–6). Other eight totally unrelated proteins presenting expanded CAG/polyQ repeats have been involved in neurodegenerative diseases (CAG/polyQ diseases) (7). Thus, the polyQ expansion is likely to confer a gain of neurotoxic function(s) to these mutant proteins. Indeed, the polyQ tract induces the acquisition of aberrant protein conformation (misfolding) to the host proteins making them prone to aggregate. Misfolded proteins affect the protein quality control (PQC) system functioning and, in SBMA, this event occurs in the cells expressing high levels of ARpolyQ. In fact, AR is abundantly expressed in motoneurons located in the anterior horns of the spinal cord and in the brain stem, as well as in sensory neurons of the dorsal root ganglia. These neurons degenerate in SBMA leading to atrophy of bulbar, facial and limb muscles, and in sensory function alterations (8–14). Also, non-neuronal cells, like the motoneuron-controlled skeletal muscle cells, are directly affected by mutant protein toxicity. Indeed, even if originally classified as a typical MND, due to the relevant involvement of muscle tissue, SBMA has been reclassified as neuromuscular disease (15–26). The involvement of the muscle cells is rather complex. The atrophy of muscle cells may result from the loss of innervation arising from affected motoneurons and/or may be a direct consequence of ARpolyQ proteotoxicity on skeletal muscle cells. In fact, like spinal cord motoneurons and dorsal root ganglia neurons, also skeletal muscle cells are post-mitotic cells highly sensitive to the presence of misfolded species of ARpolyQ (27). Several evidences obtained initially in SBMA animal models support the direct involvement of muscle in the pathogenesis of SBMA, since the inhibition of AR production selectively in muscle correlates with an amelioration of the phenotype in mice; this notion has been proposed to be valid also in SBMA patients (24, 28, 29). Even human wild type AR (wtAR) overexpressed in mouse skeletal muscle induces several alterations normally observed in SBMA (16, 18, 30, 31). In addition, the downregulation of ARpolyQ levels specifically in skeletal muscle, by mean of antisense oligonucleotides (ASOs), results in prolonged survival in different SBMA mouse models, proving ARpolyQ direct action on muscle (25, 26, 32, 33); also, the restricted overexpression of ARpolyQ in muscle cells determines a delay of the SBMA onset in mouse models. Moreover, muscle samples from SBMA patients show dysregulation of several important pathways such as mitochondrial turnover, or the neuromuscular transmission at birth with an increased expression of the neonatal isoform of acetylcholine receptor (34). A major aspect of SBMA is that castration completely rescues SBMA phenotype in male mice, ascribing SBMA onset to testosterone activation of ARpolyQ (35–38), even if some early symptoms could appear in an androgen-independent manner (39). Muscle is the typical direct target of the anabolic androgenic activity of the AR (40, 41), and, thus, testosterone-triggered ARpolyQ toxicity may sensitize skeletal muscle cells to “toxic” ARpolyQ conformations, which cause ARpolyQ aggregation. In addition, testosterone induces the translocation of misfolded ARpolyQ into the nucleus where the protein exerts most of its toxicity (42, 43). These aggregates may not be toxic *per se* (44), but their presence in cell environment can lead to many cellular dysfunctions. However, misfolded ARpolyQ are likely to be formed soon after the release from HSPs which occurs in the cell cytoplasm, and there is the possibility to clear them as soon as they are formed prior to their migration into the cell nuclei. A typical cytoplasmic degradative process which may prevent misfolded ARpolyQ accumulation, or aberrant nuclear migration, is autophagy. Unfortunately, in the cytoplasm the ARpolyQ protein may block the autophagic flux due to misfolded proteins overload (45–51). Autophagy is considered one of the most important degradative system in cells, since its impairment in neurons leads to their death (52, 53). Autophagy is based on the formation of autophagosomes that entrap the waste material which will be then degraded when autophagosomes fuse with lysosomes (54). Indeed, by using trehalose, a well-known activator of the autophagy master regulator transcription factor EB (TFEB) (55, 56), to restore a normal autophagic flux in SBMA neuronal models, we found an improved clearance of misfolded ARpolyQ and the prevention of its aggregation (49, 51, 56), particularly in motoneuron (57–59).

The importance of a functional autophagy flux in SBMA is also sustained by several studies performed in animal and cell models of SBMA (50, 60, 61). In particular, autophagy is dysregulated in muscles of AR113Q knock-in SBMA mice (19, 22, 26), and this dysregulation includes alteration of TFEB, and its physiological antagonist ZKSCAN3 (22), as well as TFEB-target genes (coding for LC3, VPS11, VPS18 and LAMP1), both in mice and in patients (22). Notably, the inhibition of BECN1/Beclin1-mediated autophagy activation in AR113Q knock-in SBMA mice reduces skeletal muscle atrophy, extends survival and improves the phenotype, while over-activation of autophagy worsens phenotype (19). Thus, a crucial point when considering autophagy is that its levels of activation must be finely tuned, and, thus, any autophagy stimulator must be able to prevent accumulation of harmful material preserving cell's functionality. In this scenario, autophagic clearance of ARpolyQ in skeletal muscle, and how this is related to alternative degradative systems could have a high relevance. However, autophagy works in conjunction with the ubiquitin-proteasome system (UPS) in the removal of misfolded ARpolyQ, and its aggregated forms.

Interestingly, skeletal muscles of SBMA mice also display a high activation of *Tgfb1, Ppargc1a, Pax7, Myog*, E2-ubiquitin ligase *Ube2q1*, but not of *Myod*, and of two E3-ligases (*Trim63/Murf*-1 and *Cul3*). We found that the skeletal muscle of SBMA mice are characterized by a dramatic perturbation of several components of the autophagic pathways (*Becn-1, Atg10, Sqstm1/p62, Lc3*), particularly those involved in the peculiar autophagic process now recognized as chaperone-assisted selective autophagy (CASA) (56, 61–72), like the CASA complex components: the small heat shock protein (HSP) B8 and BAG3, which in cooperation with the co-chaperone BAG1 control the correct routing of misfolded proteins to clearance (61). The *Hspb8, Bag3* and *Bag1* gene are all iper-induced in skeletal muscle of SBMA mice, and the *Bag3:Bag1* ratio is increased in these muscles (73). Of note, the equilibrium between UPS and autophagy is critical to maintain the regular misfolded ARpolyQ clearance in SBMA (61). The molecular players regulating the equilibrium that re-routes substrates to UPS or autophagy are BAG1, which mediates UPS clearance of clients, and BAG3 which controls autophagic clearance of clients (46, 48, 49, 61, 68, 72, 74). BAG3 interacts (in a 2:1 ratio) with HSPB8, and the complex reduces ARpolyQ aggregation, by enhancing its solubility and clearance acting as an autophagy facilitator (49, 61). In this process HSPB8/BAG3 complex needs to interact with HSC70/CHIP dimer and the client misfolded protein, allowing its ubiquitination for SQSTM1/p62-mediated insertion into autophagosomes (63, 65). Only few studies aimed to unravel the involvement of the HSPB8-BAG3 and BAG1 systems in SBMA skeletal muscle (73), but the identification of specific autophagy related molecular target might represent a therapeutic valuable strategy for counteracting ARpolyQ toxicity (73). Notably, both *HSPB8* and *BAG3* mutations have been linked to neuromuscular disorders suggesting that they may be deeply involved in the regulation and in the control of the proteotoxic response of muscle cells (70, 71, 75–79).

For all these reasons, in this study, we have provided an extensive characterization of the autophagic activation, the role of the CASA complex and the HSPB8/BAG3 machinery as well as of the BAG1 co-chaperone in the PQC system response in a SBMA muscle cellular model.

## Materials and Methods

### Chemicals

Testosterone; Z-Leu-Leu-Leu-al or MG132; Bafilomycin A1 from *Streptomyces griseus*; D-(+)-Trehalose dihydrate were all obtained from Sigma-Aldrich (St. Louis, MO, USA).

### Cell Cultures, Treatments, and Transfection

Immortalized mouse myoblast C2C12, stably transfected, respectively, with cDNA encoding the human full length wt AR (with 24 Qs = ARQ24), or the mutant AR (with elongated polyQ of 100 Qs = ARQ100), were obtained by infection with the Lentiviral vector #945.PCCL.sin.cPPT.SV40ployA.eGFP.minCMV.hPGK.deltaLN-GFR containing the human cDNA encoding the ARQ24 or the ARQ100 (s-myoblasts) (80). After transfection, cells were sorted using the GFP fluorescence to identify positive cells. Sorted cells of both lines were cultured with DMEM high glucose medium (Euroclone, Pero, MI, Italy) supplemented with 1 mM glutamine (Euroclone), (30 μg/mL) penicillin [SERVA, Electrophoresis GmbH, Heidelberg, Germany (64μg/mL)] streptomycin (SERVA), and 10% charcoal-stripped fetal bovine serum (CS-FBS) (GIBCO, Thermo Scientific Life Sciences Research, Waltham, MA, USA), to deplete hormones contained in the serum. Basal C2C12 cells were grown in medium containing unstripped serum. Testosterone was added in presence of CS-FBS. Cells were regularly maintained at 37°C, with 5% CO_2_, and propagated after trypsin (Euroclone) dissociation as previously described (81). Cells were treated with testosterone (10 nM) for 48 h (ethanol was used as control); MG132 (10 μM) for 16 h (DMSO was used as control); Bafilomycin A1 from *Streptomyces griseus* (100 μM) for 16 h (DMSO was used as control); D-(+)-Trehalose dihydrate (100 mM) for 48 h (diluted directly in the culture medium), as detailed in figure legends.

Lipofectamine® 2000 Transfection Reagent (Thermo Scientific Life Sciences Research) was used to transfect cells, using 2 μL for transfecting 1 μg of DNA. 12-well plates were transfected with 1 μg of DNA, while 24-well plate were transfected with 0.5 μg of DNA. After 5 h, medium was replaced.

The following plasmids were used: p5HBhARQ112 (kindly provided by Dr. A.P. Lieberman, University of Michigan, Ann Harbor) here referred as ARQ112; pARQ16ΔHA, pARQ112ΔHA (kindly provided by Dr. Diane Merry, Thomas Jefferson University, Philadelphia); pCI-HSPB8 encoding human HSPB8, pCI-neo-6xHisBAG3 encoding the full-length form of human BAG3 and pCDNA/HA-BAG1 encoding the human BAG1, were all kindly provided by Prof. H. H. Kampinga (Groeningen University, Groeningen, The Netherlands); pEGFP-N1 (Clontech-Takara Bio, Saint-Germain-en-Laye, France) was utilized to determine transfection efficiency.

### PBS and NP-40 Protein Extraction

PBS extracts: cells were plated in 12-well plate at a density of 65,000 cells/well, and the day after plating, cells were transfected and/or treated. At the end of the experiment, cells were harvested, centrifuged (100 x g; 6 min; 4°C), and diluted in 60μL of PBS (Euroclone) added of protease inhibitor cocktail (Sigma-Aldrich), containing individual components including AEBSF at 104 mM, Aprotinin at 80 μM, Bestatin at 4 mM, E-64 at 1.4 mM, Leupeptin at 2 mM and Pepstatin A at 1.5 mM. After slight sonication using Bandelin Sonoplus Ultrasonic Homogenizers –HD 2070, protein content of each sample was quantified by bicinchoninic acid (BCA) assay (Euroclone).

NP-40 extracts: cells were plated in 6-well plate at a density of 130,000 cells/well. After treatments, cells were harvested, centrifuged (100 x g; 6 min; 4°C), and diluted in 65 μL in NP-40 extraction buffer (composition: 150 mM NaCl (Sigma-Aldrich); 20 mM TrisBase (Sigma-Aldrich); 0.5% Nonidet P-40 (NP-40) (Sigma-Aldrich); 1,5 mM MgCl2 (Sigma-Aldrich); 3% Glycerol (Sigma-Aldrich), pH 7.4), added of protease inhibitors [complete EDTA-free Tablet 25X (Sigma-Aldrich)], and 1 mM 1,4-Dithiotreitol (Sigma-Aldrich). Cells were lysed by passage in syringe (27 gauges). Samples were then centrifuged (16,000 x g; 15 min; 4°C). Supernatants were transferred in new tubes, and the pellets were rinsed in 65 μL of NP-40 extraction buffer. Protein content of the NP-40 soluble fraction was quantified by BCA assay (Euroclone). The insoluble fraction was sonicated following the same protocol described above.

### Filter Retardation Assay

Filter retardation assay (FRA) was performed using Bio-Dot SF Microfiltration Apparatus (Bio-Rad, Hercules, CA, USA). Six micrograms of both PBS and NP-40 soluble extracts were loaded on a cellulose acetate membrane with pores of 0.22 μm. For NP-40 insoluble extracts, the amount to be loaded was calculated as equal volume to NP-40 soluble extracts. After loading, the samples onto the cellulose acetate membrane, vacuum was applied at the apparatus and protein suspension was filtered. Proteins were fixed at the membrane using a 20% methanol solution, and the membrane was incubated for 1 h at RT in blocking solution [5% non-fat dried milk (Euroclone) in TBS-T 1X]. The membrane was then incubated with rabbit polyclonal anti-AR antibody (AR-H280, Santa-Cruz, sc-13162; dilution 1:1,000 in blocking solution) for at least 2 h at RT. After two washes with 1X TBS-T, the membrane was incubated for 1 h at RT with goat anti-rabbit HRP-conjugate secondary antibody (Santa Cruz Biotechnology, sc-2004; dilution 1:5,000 in 1X TBS-T). After three washes in 1X TBS-T signal was revealed with Clarity™ Western ECL Blotting Substrate (Bio-Rad) and optical density was acquired by ChemiDoc XRS System (Bio-Rad). Results were analyzed using Prism 5.0. Sample variations were related alternatively to ARQ24 (EtOH) or ARQ100 (EtOH). Statistical differences were obtained applying the two-way ANOVA test followed by Bonferroni *post-hoc* test. Each experiment was replicated three times, and each bar represents mean ± SEM of three independent biological replicates.

### Western Blot Analysis

Western blot experiments were performed using 10% polyacrylamide gels. To visualize AR protein, 15 μg of each PBS extract or 30 μg of each NP-40 soluble and insoluble extracts were loaded on gels. After electrophoresis, proteins were transferred over night at 4°C on nitrocellulose membrane (Bio-Rad). Membrane was then incubated 1 h at RT in blocking solution, and then overnight at 4°C with primary antibody diluted in blocking solution (5% dried non-fat milk (Euroclone) in 1X T-BST). After two washes with 1X TBS-T, the membrane was incubated 1 h at RT with secondary antibody diluted in 1X TBS-T. Signal was revealed using Clarity^TM^ Western ECL Blotting Substrate (Bio-Rad) and images were acquired by ChemiDoc XRS System (Bio-Rad) as described for FRA. The following primary antibody were used: rabbit polyclonal AR-H280 antibody (Santa-Cruz Biotechnology, sc-13162; dilution 1:1,000) rabbit polyclonal anti-LC3-B antibody (Sigma-Aldrich, L8918; dilution 1:1,000), rabbit polyclonal anti-p62/SQSTM1 antibody (Abcam, Cambridge, UK, ab91526; dilution 1:3,000), home-made rabbit polyclonal anti-HSPB8 (kindly provided by Dr. Landry, Centre of Recherche Cancerologie, University of Laval, Canada; dilution 1:2,000), rabbit polyclonal anti-GAPDH (Santa Cruz Biotechnology, sc-32233; dilution 1:1,000), goat polyclonal anti-ACTIN (Santa Cruz Biotechnology, sc1615; dilution 1:1,000), mouse monoclonal anti-α-TUBULIN (Sigma-Aldrich, T6199; dilution 1:3,000). The following secondary antibodies were used: goat anti-rabbit HRP-conjugate secondary antibody (Santa Cruz Biotechnology, sc-2004; 1:10,000), goat anti-mouse HRP-conjugate secondary antibody (Santa Cruz Biotechnology, sc-2005; 1:10,000), donkey anti-goat HRP-conjugate secondary antibody (Santa Cruz Biotechnology, sc-2020; 1:10,000).

### Immunostaining and Confocal Microscope Analysis

Cells were seeded on coverslips at a density of 25,000 cells/well (in 24-well plate), and the day after plating were transfected and/or treated. After treatments, cells were fixed at 37°C for 25 min using a solution 1:1 of 4% paraformaldehyde (Sigma-Aldrich) in PB 0.2 M [a solution made of KH2PO4 (0.06M) and Na2HPO4 (0,26M)] and 4% sucrose (Sigma-Aldrich) in PB 0.2 M. Then, fixing solution was removed and iced methanol was added for 10 min to complete the fixation. Cell permeabilization was performed using a solution of 0.2% TRITON X100 (Sigma-Aldrich) followed by incubation for 1 h in blocking solution (5% dried non-fat milk in 1X T-BST). Incubation with the primary antibody was kept o/n at 4°C. Incubation with the fluorescent-tagged secondary antibody was preceded by three washes with PBS, to remove the excess of primary antibody. Nuclei were stained with DAPI (Sigma-Aldrich). The following primary antibodies were used: rabbit polyclonal AR-H280 antibody (Santa-Cruz Biotechnology, sc-13162; dilution 1:500), rabbit polyclonal anti-LC3 antibody (Sigma-Aldrich, L8918; dilution 1:500), rabbit polyclonal anti-p62/SQSTM1 antibody (Abcam, ab91526; dilution 1:500). The following secondary antibodies were used: goat anti-rabbit Alexa 594 (Life technologies, Thermo Scientific, A-11012; dilution 1:1,000). All the primary and secondary antibodies were diluted in blocking solution. Coverslips were mounted on a glass support using MOWIOL and images were acquired using an Axiovert 200 microscope (Zeiss Instr., Oberkochen, Germany) combined with a Photometric Cool-Snap CCD camera (Ropper Scientific, Trenton, NJ, USA) or using Eclipse T*i*2 (Nikon, Netherlands) confocal microscope equipped with A1 plus camera (Nikon) and processed with the NIS-Elements software (Nikon) or using LSM510 Meta system confocal microscope (Zeiss, Oberkochen, Germany) and processed with the Aim 4.2 software (Zeiss).

### Real Time PCR

Cells were plated in 6-well plate at a density of 130,000 cells/well, and the day after plating were transfected and/or treated. At the end of the experiment, cells were harvested, centrifuged (100 x g; 6 min; 4°C) and lysed using TRI Reagent (Sigma-Aldrich). RNA was extracted following manufacturer instructions and quantified using NanoDrop 2000 spectrophotometer (Thermo Scientific). After DNA removal using DNase I (Sigma-Aldrich), 0.5 μg of the total mRNA was reverse-transcribed using High-Capacity cDNA Archive Kit (Thermo Scientific). mRNA levels were assayed using iTaq SYBR Green Supermix (Bio-Rad) on CFX 96 Real-Time System (Bio-Rad). All results were normalized to *RplP0* used as control. All the primers used were obtained by Eurofins Genomics, sequences of primers have been previously reported (73). The following primers were newly designed: *Vcp* FW- 5′-TGCCATCCTAAAAGCCAATC-3′ RV- 5′-TCAGCTCCAGAAAAGCCATT-3′.

### Statistical Analysis

Statistical analysis has been performed by using Student's *t*-test to compare two groups and analysis of variance (ANOVA) to compare three or more groups. Two-Way ANOVA was used to compare the effect of two independent variables. Analyses were performed with the PRISM (version 5) software (GraphPad Software).

## Results

### AR Aggregation in Muscle Cells

**Figure 1:**
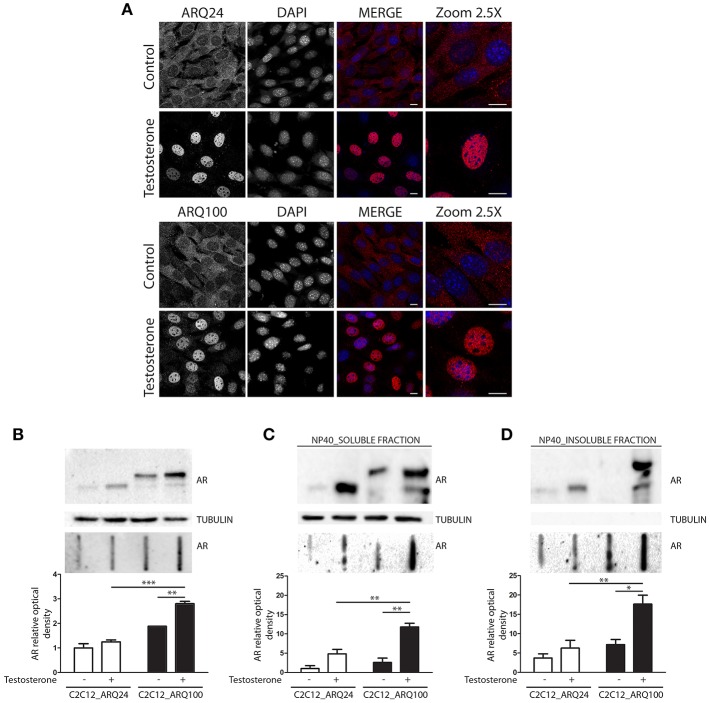
Characterization of the cellular model. **(A)** Immunostaining for AR. Nuclei staining: DAPI. 60X magnification. Confocal microscope: Eclipse T*i*2 (Nikon). Scale bar = 10 μm **(B)** WB (upper inset) and FRA (middle inset) of PBS extracts. Optical densitometry quantification of FRA (lower inset). ***p < 0.001 vs. ARQ24+T; **p > 0.01 vs. ARQ100-T. **(C)** WB (upper inset) and FRA (middle inset) of NP-40 soluble extracts. Optical densitometry quantification of FRA (lower inset). (**p < 0.01 vs. ARQ24+T or vs. ARQ100-T). **(D)** WB (upper inset) and FRA (middle inset) of NP-40 insoluble extracts. Optical densitometry quantification of FRA (lower inset). (**p < 0.01 vs. ARQ24+T; *p < 0.05 vs. ARQ100-T). Two-way ANOVA followed by Bonferroni *post-hoc* test was used. Each experiment was independently replicated three times. Graphs show quantification of three independent biological samples (*n* = 3).

Here we used immortalized C2C12 myoblasts, that are widely used as model to mimic muscle cells in culture; this cell line has been infected with viral vectors expressing ARQ24 or ARQ100, subcloned and stabilized in culture (s-myoblast). We initially performed a characterization of the AR biochemical properties in s-myoblasts to assure that the viral expression of this protein was retained even after several passages in culture. Immunofluorescence (IF) analysis showed that ARQ24 and ARQ100 have similar fluorescence intensity, and are both localized in the cell cytoplasm in basal conditions; as expected, upon testosterone treatment they both translocated into the nucleus (Figure 1A) and signal intensity also increased upon testosterone treatment. No visible aggregates or inclusions were seen by IF in C2C12-ARQ100 cell line, even after testosterone treatment. Western blot (WB) correctly showed ARQ24 with a higher SDS-PAGE motility than ARQ100, because of the presence of the polyQ tract of different length which results in different molecular weights (MW) of the two AR proteins. Moreover, both ARQ24 and ARQ100 expression was stabilized by testosterone treatment which also induced a mild upshift of the band possibly linked to AR phosphorylation during activation process (27, 82) (Figure 1B, upper panel). No high MW (HMW) forms were observed in the stacking gel in all the tested conditions (not shown), suggesting that the AR does not form SDS-resistant insoluble species in s-myoblasts. A low intensity band, possibly related to ARpolyQ fragmentation (or to the endogenous mouse AR) appeared to be mildly increased in ARpolyQ testosterone-treated samples. Interestingly, filter retardation assay (FRA) showed that, after testosterone exposure, ARQ100 formed aggregated species that can be retained on cellulose acetate membrane (with size exclusion of 0.22 μm) (***p < 0.001 vs. testosterone-treated C2C12-ARQ24; **p < 0.01 vs. untreated C2C12-ARQ100) (Figure 1B, lower panel). In this analysis, ARQ24 immunoreactivity was very low even after its activation with testosterone. To better characterize the ARQ100 aggregated species identified in FRA (but not visible in IF), we performed a detergent fractionation assay using NP-40 extraction on cell lysates. In WB, we found that large amounts of ARQ24 and ARQ100 were present in the NP-40 soluble fraction of testosterone activated ARs samples, which were considerably higher than those found for the corresponding untreated ARs samples (Figure 1C, upper panel). This confirmed testosterone stabilization of AR protein (83). Of note, in the NP-40 insoluble extracts we found a much more abundant amounts of testosterone-treated ARQ100 compared to testosterone-treated ARQ24, and to untreated controls (Figure 1D, upper panel). Using FRA analysis, we found that testosterone treatment triggered the formation of NP-40 soluble, and NP-40 insoluble aggregates retained on the cellulose acetate membrane of ARQ100, while these species were not formed by ARQ24 (**p < 0.01 vs. ARQ24 cell line) (Figures 1C,D, lower panels). Despite these data, we found no differences in cell viability, or cell survival in cells expressing ARQ24 or ARQ100, even after testosterone treatment (data not shown), suggesting that s-myoblasts are not sensitive to ARpolyQ toxicity.

Collectively these data suggest that testosterone induces the formation of ARQ100 aggregates detectable in FRA. These species are present both in PBS extracts and in NP-40 soluble, and insoluble extracts. Surprisingly, no aggregates were observed in IF. It might be possible that their size is lower than the detection sensitivity of IF as in the case of small oligomeric species.

### The Impact of the Modulation of the Protein Quality Control System on AR Aggregation in Muscle Cells

**Figure 2:**
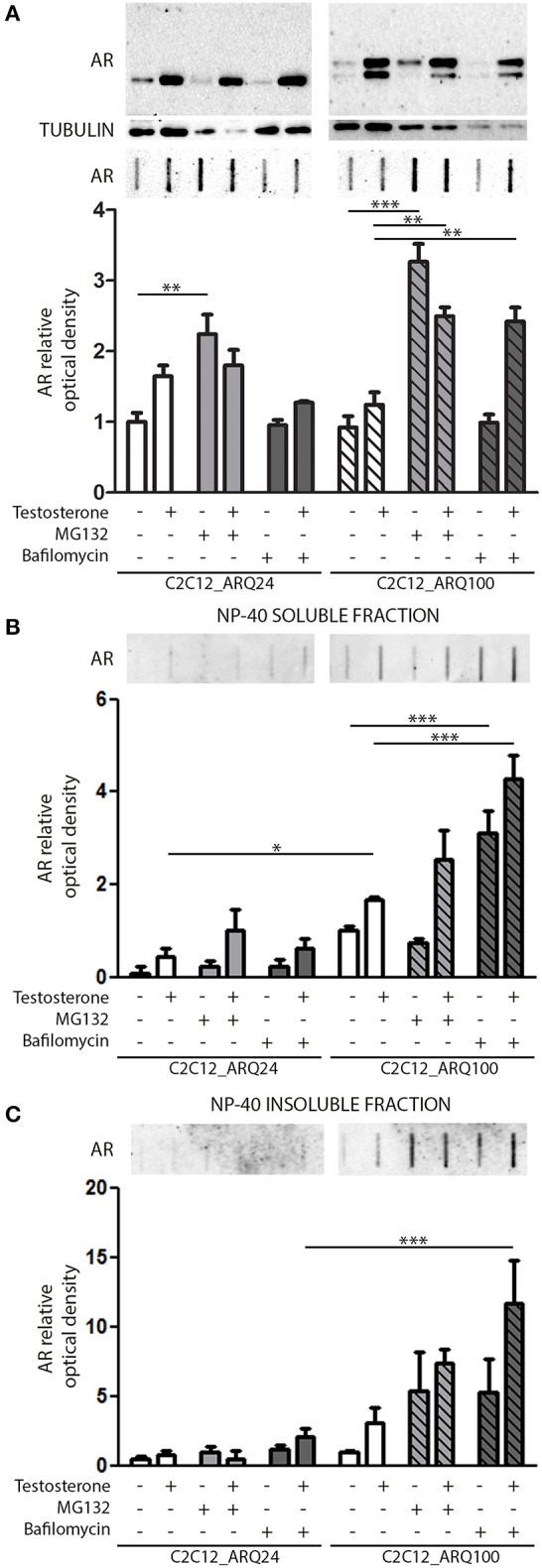
Degradative systems involvement. **(A)** WB (upper inset) and FRA (middle inset) of PBS extract of cells treated with testosterone, MG132 and bafilomycin A1. Optical densitometry quantification of FRA (lower inset). (**p < 0.01; ***p < 0.001 vs. relative control conditions –T/+T). **(B)** FRA (upper inset) of NP-40 soluble extracts of cells treated with testosterone, MG132 and bafilomycin A1. Optical densitometry quantification of FRA (lower inset). (***p < 0.001 vs. relative control conditions –T/+T; *p < 0.05 vs. ARQ24 -T). **(C)** FRA (upper inset) of NP-40 insoluble extracts of cells treated with testosterone, MG132 and bafilomycin A1. Optical densitometry quantification of FRA (lower inset) (***p < 0.001 vs. relative control conditions +T). For each panel, FRA images derive from the same membranes with identical exposure time to permit direct comparison of wtAR and ARpolyQ levels. Two-way ANOVA followed by Bonferroni *post-hoc* test was used. Each experiment was independently replicated three times. Graphs show quantification of three independent biological samples (*n* = 3).

We next investigated which degradative pathway is specifically responsible for ARQ24 and ARQ100 degradation in s-myoblasts by inhibiting the UPS or autophagy, using MG132 or bafilomycin A1, respectively. Proteasome inhibition resulted in an increase of the accumulation of the total amounts of ARQ24 in FRA, which is normally processed via this degradative pathway (47) (Figure 2A, lower inset), showing that a high concentration of wtAR inside cells (associated to its impaired clearance) could lead to its accumulation in HMW species. In s-myoblasts, proteasome inhibition resulted in a dramatic increase of the accumulation of mutant ARQ100 in FRA independently from its activation, as we already reported for immortalized motoneurons (47). We performed detergent fractionation assay, and we found no difference in the levels of ARQ24 species after proteasome inhibition (Figures 2B,C), suggesting a variability in the response of normal (ARQ24) cells to UPS inhibition. Conversely, both NP-40 soluble and insoluble testosterone-induced ARQ100 aggregates, which are retained on cellulose acetate membrane, were increased after proteasome inhibition (Figures 2B,C, lower insets).

With regards to autophagy, we found no involvement of this pathway in the clearance of the wtAR (ARQ24) in s-myoblasts, while the perturbation of autophagosome and lysosome fusion with bafilomycin A1 resulted in a robust increase of PBS soluble form of ARQ100 in presence of testosterone (Figure 2A) in FRA. Bafilomycin A1-mediated inhibition of autophagy resulted also in a dramatic increase of both ARQ100 NP-40 soluble and insoluble species, independently from testosterone treatment (Figures 2B,C).

These data suggest that in s-myoblasts proteasome is the main mediator of the clearance of both wt and mutant AR, while autophagy appears to be predominantly involved in the clearance of the mutant ARpolyQ.

**Figure 3:**
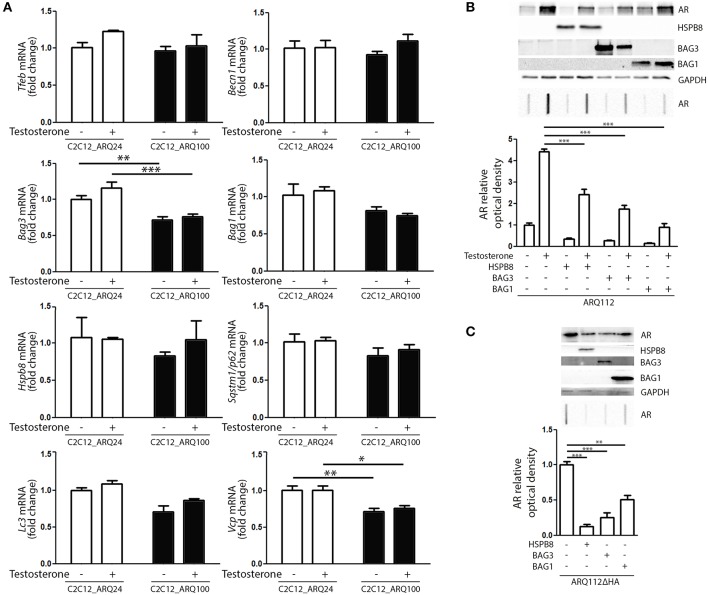
PQC activation and role against AR accumulation. **(A)** RT-qPCR of PQC system related genes performed on C2C12_ARQ24 and C2C12_ARQ100. *p < 0.05; **p < 0.01; ***p < 0.001 vs. ARQ24 in the same conditions. Graphs show quantification of four independent biological samples (*n* = 4). **(B)** WB (upper inset) and FRA (middle inset) of C2C12 transiently transfected with p5HBhARQ112 and co-transfected with plasmids coding for HSPB8, BAG3 and BAG1. Optical densitometry quantification of FRA (lower inset). (***p < 0.001 vs. relative control conditions +T). **(C)** WB (upper inset) and FRA (middle inset) of C2C12 transiently transfected with pARQ112ΔHA and co-transfected with plasmids coding for HSPB8, BAG3 and BAG1. Optical densitometry quantification of FRA (lower inset). (**p < 0.01; ***p < 0.001 vs. relative control conditions pcDNA3). Each experiment was independently replicated three times. Graphs show quantification of three independent biological samples (*n* = 3).

Next, we evaluated whether the presence and activation of mutant ARpolyQ have an impact on the expression of genes involved in the PQC system. We found no variation in the expression of *Tfeb, Becn1, Bag1, Hspb8, Sqstm1/p62, Lc3* in all conditions tested (Figure 3A). We found that the expression of mutant ARpolyQ correlated with a reduction in the expression of *Bag3* and *Vcp* (another autophagy associated proteins found to be involved in motoneuron diseases), but these changes were not linked to the presence of testosterone (Figure 3A).

We then analyzed whether the levels of ARpolyQ species entrapped in FRA could be modulated by the overexpression of components required to route misfolded proteins to either UPS or autophagy. The data shown in Figure 3B indicate that the formation of testosterone-induced aggregated species of mutant ARpolyQ in transiently transfected C2C12 (ARQ112 HMW aggregates) can be counteracted by the overexpression of HSPB8 and BAG3. These two proteins are essential components of the CASA complex, which delivers misfolded proteins to the microtubule organization center where aggresomes are formed before their engulfment into nascent autophagosomes. Notably, both HSPB8 alone, and BAG3 alone preserve their pro-autophagic activity even if the CASA complex required both proteins in association with HSP70 and CHIP. This suggests that may be both considered limiting factor for the CASA complex activity. Interestingly, also the overexpression of BAG1 resulted in a great reduction of the accumulation of testosterone-induced aggregated species of mutant ARpolyQ measured in FRA (Figure 3B). It must be noted that BAG1 exerts its activity by preventing HSP70 and CHIP to become part of the CASA complex (61, 84–86), thus routing misfolded proteins to UPS degradation as an alternative to autophagy. Since, it has been demonstrated that testosterone induces the formation of ARpolyQ aggregates via the generation of a N-terminal caspase-3 cleaved fragment containing the polyQ stretch, which is highly prone to aggregate, we wanted to test whether the routing system may also be involved in the removal of this highly neurotoxic AR species. The results (Figure 3C) clearly demonstrated that both the overexpression of HSPB8 and BAG3, as well as that of BAG1, are capable to revert the accumulation in FRA of HMW aggregates of a highly neurotoxic caspase-3 released N-terminal fragment of ARpolyQ ARQ112ΔHA (87–90).

Collectively, these data suggest that, by modulating specific components of the PQC system, the ARpolyQ and its highly neurotoxic aggregate-prone caspase-3 released fragment can be eliminated from muscle cells using both the proteasome and the autophagy system, when they are still normal and functioning as in our cell line [ARQ100 does not greatly affect proteasome and autophagy machinery (Figure 3A)].

### Pharmacological Induction of the Autophagic System Reduces ARpolyQ Accumulation and Aggregation

**Figure 4:**
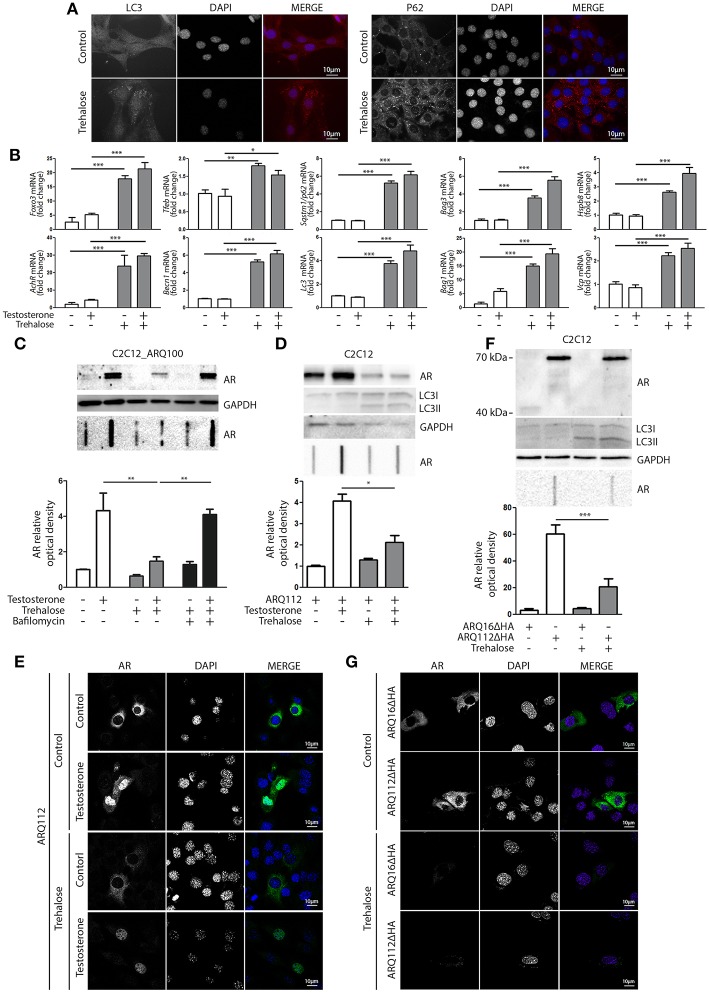
Trehalose activates autophagy and reduces ARpolyQ accumulation **(A)** Immunostaining for LC3 (left inset) and p62 (right inset) of C2C12_ARQ100 in presence of trehalose. Nuclei staining: DAPI. Magnification: 63X. Microscope: Axiovert 2000 **(B)** C2C12_ARQ100 treated with trehalose. RTqPCR of PQC system related genes. (**p < 0.01; ***p < 0.001 vs. relative untreated control). **(C)** C2C12_ARQ100 treated with trehalose and bafilomycin A1. WB (upper inset) and FRA (middle inset) of PBS extracts of Optical densitometry quantification of FRA (lower panel) (**p < 0.01 vs. relative untreated control; ***p < 0.001 vs. trehalose +T). **(D,E)** C2C12 transiently transfected with p5HBhARQ112 and treated with testosterone and trehalose. **(D)** WB (upper inset) and FRA (middle inset). Optical densitometry quantification of FRA (lower inset). *p < 0.05 vs. relative control conditions +T. **(E)** Immunostaining for AR. Nuclei staining: DAPI. Magnification: 63X. Microscope: confocal LSM510 Zeiss **(F,G)** C2C12 transiently transfected with pARQ16ΔHA or pARQ112ΔHA and treated with trehalose. **(F)** WB (upper inset) and FRA (middle inset). Optical densitometry quantification of FRA (lower inset). **p < 0.01 vs. relative control conditions +T. **(G)** Immunostaining for AR. Nuclei staining: DAPI. Magnification: 63X. Microscope: confocal LSM510 Zeiss. Each experiment was independently replicated three times (*n* = 3). Graphs show quantification of three independent biological samples (*n* = 3).

Based on these data, we hypothesized that compounds capable of activating autophagy may serve to enhance the ARpolyQ clearance from muscle cells. We use a well-known autophagy activator, trehalose, which acts in a mTOR-independent manner. We recently described that trehalose causes a transient lysosomal damage, which in turn activates TFEB and, consequently, promotes autophagosome and lysosome assembly and fusion (56). We found that in s-myoblasts, trehalose retained its capability to activate autophagy, as demonstrated by the conversion of LC3 from its LC3-I diffuse form to the LC3-II lipidated form associated to autophagosomes in its punctate status (Figure 4A, left insets) or by the relocalization of SQSTM1/p62 into p62 bodies (Figure 4A, right insets). These data were also corroborated by the mRNA expression analysis showing that trehalose induced the *de novo* expression of several pro-autophagic genes, including *Foxo3, Tfeb, Becn1, Bag3, Bag1, Hspb8, Lc3, Sqstm1/p62, Vcp*, and *AchR* (Figure 4B).

The effects of trehalose were then tested on the accumulation of ARQ100 in s-myoblasts and the data showed that this autophagy activator reduced the levels of monomeric soluble ARQ100, and fully counteracted the accumulation of testosterone-induced HMW aggregated species of ARQ100 (Figure 4C). Trehalose effect on mutant ARpolyQ was fully blocked by bafilomycin A1, proving that its pro-degradative activity is mediated by autophagy. The effects of trehalose on ARpolyQ clearance were also maintained when the mutant protein was transiently overexpressed in basal C2C12, since this autophagy activator significantly reduced both the monomeric soluble ARQ112 evaluated in WB (Figure 4D, upper inset), and testosterone-induced aggregated species of ARQ112 evaluated in FRA (Figure 4D, lower inset). Trehalose activation of autophagy was tested in basal C2C12 expressing ARQ112 only by LC3 conversion in WB, assuming that the effects observed by RT-qPCR and IF in s-myoblasts expressing ARQ100 were recapitulated also in basal C2C12, and the data confirmed that trehalose acts as a potent autophagy inducer as previously shown (51, 56, 91, 92). Importantly, testosterone-dependent ARQ112 inclusions observed in IF (Figure 4E) were found to be fully degraded after trehalose treatment. Finally, we found that activation of autophagy with trehalose counteracted the accumulation also of the aggregated species of fragmented ARQ112ΔHA retained in FRA (Figure 4F), and the ARQ112ΔHA inclusions evaluated in IF (Figure 4G).

## Discussion

In this study, we characterized the biochemical behavior of the mutant ARpolyQ in s-myoblasts, and we compared it to the one of the wtAR. We found that, in these cells, the mutant ARpolyQ does not form inclusions visible by microscopy, or detectable by immunoblotting as SDS-insoluble aggregates in WB, even after its testosterone activation. Instead, we found that ARpolyQ generated testosterone-inducible aggregated species readily detectable in FRA, which were resistant to NP-40 solubilization. Notably wtAR insoluble species were detected only in the PBS resistant fraction, but not in NP-40 soluble or insoluble fractions, suggesting that even if formed they remain largely soluble, while those formed by mutant ARpolyQ becomes detergent-insoluble. Both the wtAR and the mutant ARpolyQ are processed via the proteasome, while only mutant ARpolyQ is cleared by autophagy, since autophagy inhibition resulted in a robust accumulation of ARpolyQ insoluble species in FRA. Despite this, the presence of ARpolyQ was insufficient to trigger an autophagic response, since no variation were found in the expression of classical autophagy related genes (e.g., *Tfeb, Becn1, Bag1, Hspb8, Sqstm1/p62, Lc3*) even after testosterone treatment. Of note, we found that ARpolyQ activation in s-myoblasts correlated with a reduced expression of two pro-autophagic proteins such as BAG3 and VCP. Mutations in *BAG3* and *VCP* genes are responsible for late onset degenerative diseases affecting skeletal muscle (93, 94), suggesting that these proteins might play an important role in the maintenance of muscle cell homeostasis. This phenomenon is not correlated to the presence of testosterone, but still suggestive of a decreased autophagic response in s-myoblasts in presence of ARpolyQ. Overall these results point to the fact that activated ARpolyQ does not greatly affect the functionality of the PQC system in our muscle cell model. Anyway, it might be possible that the mildly reduced autophagic potential causes ARpolyQ aggregation only after testosterone activation, slowing down ARpolyQ clearance via autophagy. To facilitate the degradation of ARpolyQ, we overexpressed BAG3 or its partner HSPB8, essential components of the CASA complex, showing that they are both able to enhance ARpolyQ clearance even in presence of testosterone. Also, the overexpression of BAG1, the co-chaperone which routes the HSP70/CHIP/misfolded protein complex to UPS (95–97), exerted a similar effect on ARpolyQ clearance. Overexpression of these chaperones was effective also against aggregates formed by the N-terminal ARpolyQ fragment, physiologically formed upon testosterone treatment. Thus, the modulation of the PQC could be viewed as a potential target to ameliorate the removal of toxic ARpolyQ from our muscle cell models. Indeed, by adopting a pharmacological treatment with trehalose, which is a mTOR independent autophagy activator, we have clearly shown that the insoluble species of ARpolyQ disappeared, both using the ARQ100 in stably infected cells as well as with ARQ112 or the caspase-3 released N-terminal fragment transiently transfected in s-myoblasts. The involvement of autophagy in mediating the pro-degradative activity of trehalose in s-myoblasts was proved by the fact that treatment with bafilomycin A1 fully reverted the protection exerted by trehalose against ARpolyQ accumulation.

In our view, these data acquire particular relevance keeping in mind that SBMA, regarded for years as a MND (3, 98), has now been defined as a neuromuscular disease (25). Muscle tissue is a primary site for SBMA toxicity as muscle atrophy often precedes motoneuron loss and the onset of SBMA is rescued by specific repression of ARpolyQ in muscle cells (33, 50). Even if these studies were carried out in murine models and findings remain to be confirmed in human cell lines, they support the notion of a direct muscle involvement in SBMA onset and progression. Here, we found that autophagy activation, or facilitation, prevents ARpolyQ accumulation in our muscle cell models, suggesting that autophagy could be a specific pathway for the degradation of testosterone activated ARpolyQ insoluble species. In addition, autophagy appears to be partially impaired, making it an important target to facilitate misfolded ARpolyQ clearance in SBMA. Studies performed in SBMA mouse models indicated that at later stage of disease, autophagy is altered in skeletal muscle (22, 32, 50, 73, 99), but its role is still largely debated. One of the major problem linked to these analysis is that the specific time window in which the mice are analyzed (pre-symptomatic, symptomatic or end stage of disease) may influence the relative involvement of autophagy in response to mutant ARpolyQ due to several compensatory mechanisms triggered during muscle atrophy progression. In addition, the mouse SBMA models utilized significantly differ in term of level of protein expression, and its tissue distribution. However, the analysis of TFEB activity, measured by evaluating its target genes, showed that autophagy is enhanced in presence of ARpolyQ in muscle tissue (22). Using the same SBMA model, we also confirmed and extended the activation of TFEB-mediated autophagy (73). In addition, in the same animals, we demonstrated that, at the symptomatic stage, also the expression of genes involved in CASA-complex (e.g., *Hspb8* and *Bag3*) resulted upregulated. Thus, in skeletal muscle autophagy is activated during disease progression, and its upregulation might be an attempt to respond to ARpolyQ toxicity, or to mediate the catabolic activity induced by muscle atrophy associated to the chronic exposure to ARpolyQ (100). This may suggest that the autophagy response observed in the skeletal muscle of the SBMA mice is an adaptive mechanism related to both the presence of the misfolded ARpolyQ, and the muscle atrophy. In any case, we cannot exclude that aberrant autophagic upregulation contribute to SBMA progression. Overall, these data suggest that restoration of physiological autophagic function might represent an important therapeutic target for SBMA. Our s-myoblast model may be particularly relevant for the screening of compounds that may modulate autophagy dysregulation in muscle cells. In addition, our s-myoblast model will permit to evaluate the acute response to ARpolyQ activation by testosterone. Indeed, acute ARpolyQ expression leads to a mild autophagy response. Misfolded ARpolyQ production is insufficient to induce a *de novo* expression of all gene tested, with the exception of *Bag3* and *Vcp*. Our s-myoblast SBMA model may thus contribute to understand the different events taking place in skeletal muscle cells exposed to misfolded ARpolyQ allowing to discriminate between early and/or adaptive response. A possible limitation of this model is the fact that s-myoblasts do not show modification in cell viability induced by ARpolyQ. Despite this, they are characterized by the specific accumulation of testosterone-induced ARpolyQ NP-40 insoluble species (not detectable in the case of wtAR); these species clearly represent a biochemical form of misfolded ARpolyQ which play a role in SBMA pathogenesis recapitulating the disease phenotype. As it has been published (80, 101, 102), the formation of the insoluble ARpolyQ species, might be a valuable biomarker to follow the progression of muscle degeneration.

Importantly, the systems here described, including some chaperones and autophagy/proteasome, are highly conserved and work in a similar manner in neuronal, muscular and non-neuronal cells. Therefore, boosting them could provide protection by enhancing the clearing capacities, and maintaining protein homeostasis in different cell types affected by the disease.

In this context, targeting autophagy could be an efficient strategy to reduce the accumulation of ARpolyQ. Trehalose not only activates the basal autophagy process (e.g., TFEB activation enhanced SQSTM1/p62 and LC3 expression), but also increased the expression of key factors of the CASA-complex, like HSPB8, that probably helps in the recognition of selected cargo avoiding the uncontrolled degradation of every intracellular element.

Even if C2C12 SBMA cell model does not show a reduction in cell viability induced by ARpolyQ NP-40 insoluble species, it might be helpful to understand molecular mechanisms responsible for muscle degeneration observed in SBMA patients as it has been published in other publications (80, 101, 102). Since in this model AR NP-40 insoluble species are polyQ and testosterone-dependent, recapitulating the disease phenotype, these C2C12 cell lines could be used to co-culture skeletal muscle and motoneurons in order to study if ARpolyQ expression in myoblast can alter motoneuron functionality and viability.

Overall these results show that ARpolyQ aggregation may occur also in muscle cells, and that targeting aggregation of ARpolyQ could be beneficial in SBMA, since the permanence of inclusions in the cells could cause the damage of several pathways and the recruitment of other soluble proteins, impairing other pathways. Concluding, trehalose plays beneficial effects against ARpolyQ aggregation and autophagy appears as a valuable pathway for the degradation of insoluble ARpolyQ species. In parallel and supporting this study, there are several ongoing studies that are testing, in *in vivo* models, novel compounds that will address the PQC system, to reduce the presence of the misfolded toxic proteins.

## Data Availability

The datasets generated for this study are available on request to the corresponding author.

## Ethics Statement

This study is not subjected to ethical committee approval, since no animal and human data have been collected.

## Author Contributions

MEC and RC performed most of the experiments. VC, VF, BT, EC, and MC contributed to perform experiments and critically revised the manuscript. MG, MPi, and EM contributed to design the experiments and critically discussed the data. SC provided expertise on BAG1-BAG3 functions and revised the manuscript. MPe provided the stably infected cells and assisted in experiment design with the expertise on SBMA. PR provided expertise on trehalose experiments and critically revised the manuscript. AP supervised the entire study and wrote the paper.

### Conflict of Interest Statement

The authors declare that the research was conducted in the absence of any commercial or financial relationships that could be construed as a potential conflict of interest.
